# Adherence to the Recommendations from the Portuguese General Directorate of Health (GDH) during the COVID-19 pandemic: fear or prosocial behaviour?

**DOI:** 10.1192/j.eurpsy.2022.792

**Published:** 2022-09-01

**Authors:** C. Cabacos, A.T. Pereira, M.J. Pacheco, S. Soares, A. Manão, A. Araújo, A.P. Amaral, R. De Sousa, A. Macedo

**Affiliations:** 1 Faculty of Medicine of University of Coimbra, Institute Of Psychological Medicine, Coimbra, Portugal; 2 University of Coimbra, Faculty Of Medicine, Coimbra, Portugal; 3 Polytechnical Institute of Coimbra, Coimbra Health School, Coimbra, Portugal; 4 ACeS Baixo Mondego, Usf Coimbra Centro, Coimbra, Portugal

**Keywords:** altruism, Covid-19, fear, Empathy

## Abstract

**Introduction:**

During a public health crisis, preventive measures are essential. However, to make them effective, all citizens must be engaged.

**Objectives:**

To analyse the differential role of individual and contextual variables in the adherence to public health recommendations.

**Methods:**

1376 adults (70.5% female; mean age=35.55±14.27) completed a survey between September/2020 and May/2021 with: Adherence Scale to the Recommendations during COVID-19 (ASR-COVID19; evaluates three dimensions of adherence), Fear of Covid-19 Scale (FC19S) and Toronto and Coimbra Prosocial Behaviour Questionnaire (ProBeQ; assesses empathy and altruism).

**Results:**

Adherence did not differ between individuals with or without personal or family history of COVID-19 infection. ASR-COVID19 and all dimensions were positively correlated to ProBeQ’s altruism and empathy (from r=.32 to r=.54); FCV19S correlated positively to total adherence score and house sanitation (from r=.18 to r=.26; all p<.01). Linear regressions revealed that altruism and empathy (first model), as well as fear of Covid-19 (second model), were significant predictors of adherence; however, while the first model explained ≅28% of its variance, the second (FCV19S as independent variable) only explained ≅3%. Regression models performed in a subsample of participants with personal or family history of COVID-19 revealed that only empathy, but not altruism, was a significant predictor of adherence; in this subsample, fear was no longer a significant predictor of adherence, except for lockdown and use of teleservices.

**Conclusions:**

Based on our results, we suggest health care providers and public health campaigns should take into consideration social solidarity and altruism, as well as previous experiences, when appealing to public’s engagement in health behaviour.

**Disclosure:**

No significant relationships.

